# Impact of Chronic Rhinosinusitis on Granulomatosis with Polyangiitis Exacerbations

**DOI:** 10.3390/jcm14092962

**Published:** 2025-04-25

**Authors:** Trisha Shang, David C. Kaelber, Mohamad R. Chaaban

**Affiliations:** 1 School of Medicine, Case Western Reserve University, Cleveland, OH 44106, USA; txs479@case.edu; 2 The Center for Clinical Informatics Research and Education, The MetroHealth System, Cleveland, OH 44109, USA; dkaelber@metrohealth.org; 3 The Departments of Internal Medicine, Pediatrics, and Population and Quantitative Health Sciences, Case Western Reserve University, Cleveland, OH 44106, USA; 4 Head and Neck Institute, Cleveland Clinic, Cleveland, OH 44195, USA

**Keywords:** granulomatosis with polyangiitis, chronic rhinosinusitis, vasculitis, systemic progression

## Abstract

**Background/Objectives:** Granulomatosis with polyangiitis (GPA) is an autoimmune vasculitis, often presenting first with sinonasal symptoms diagnosed as vasculitis chronic rhinosinusitis (CRS). Patients with limited (L) GPA do not have renal involvement and often have more local sinonasal disease. Few studies have examined systemic progression in LGPA patients presenting with local sinonasal disease. Our objective was to compare GPA disease progression and activity in LGPA patients with and without CRS. **Methods:** Using the US Collaborative Network of the TriNetX platform, we conducted a retrospective study of adults with LGPA and CRS versus those without CRS. Outcomes were measured 1 month-5 years after patients met inclusion criteria. Primary outcomes were acute sinusitis, end-organ damage, and major GPA disease activity. Secondary outcomes were end-organ damage and major disease activity for each organ system and mortality. **Results:** There were *n* = 1097 in the LGPA with CRS cohort and n = 3331 in the LGPA without CRS cohort, with n = 1023 in each cohort after 1:1 propensity matching on age, gender, ethnicity, and race. We found a significantly greater risk of acute sinusitis (risk ratio: 4.80, 95% confidence interval: [2.89,7.99]), end-organ damage (2.99 [2.41, 3.70]), and major disease activity (2.41 [1.73, 3.35]) comparing patients with CRS to those without. LGPA patients with CRS had no significant difference in mortality compared to those without CRS (0.94, [0.64,1.38]). **Conclusions:** Patients with LGPA and CRS have greater risk of developing disease progression and increased organ system disease activity compared to LGPA without CRS.

## 1. Introduction

Granulomatosis with polyangiitis (GPA) is an autoimmune systemic granulomatous necrotizing inflammation of small and medium blood vessels [1]. If left untreated, there is approximately an 80% mortality rate within one year [2] and a nearly 10% mortality rate with immunosuppressants [3,4]. GPA manifests in the upper respiratory tract, lower respiratory tract, and kidneys [1]. Up to 85% of patients have rhinologic symptoms [5,6]. These symptoms include bloody discharge, crusting, epistaxis, and vasculitic chronic rhinosinusitis (CRS) [6,7,8]. GPA treatment typically involves systemic immunosuppressive agents, including cyclophosphamide, glucocorticoids, azathioprine, methotrexate, and rituximab [9]. For sinonasal management in GPA, treatment may also include saline irrigation, topical antibiotics, and topical nasal corticosteroids [10,11]. Patients with particularly severe CRS may undergo sinus surgery as second-line treatment for sinonasal disease refractory to medical management [11].

While GPA can involve the upper and lower respiratory tracts and the kidneys, limited GPA (LGPA) is a form that does not involve the kidneys. GPA disease activity can be measured with the Birmingham Vasculitis Activity Score for Wegener’s Granulomatosis (BVAS/WG). This uses pre-weighted variables for exacerbations that are considered more “major” within each organ system [12]. Patients with LGPA have lower baseline BVAS/WG and were more likely than those with severe GPA to have greater local nasal disease [13]. Furthermore, patients with LGPA have the potential to progress and cause more systemic exacerbations (organ damage), as defined by the Vasculitis Disease Index (VDI) [14]. The VDI measures the extent of end-organ damage from vasculitis. It requires at least 3 months of symptoms to be scored as damage, rather than ongoing disease activity [15].

Given that there is the potential for LGPA patients to have further systemic progression and given that otolaryngologists are faced with presentations that could be the earlier manifestations of LGPA with CRS, it is important for them to have an understanding of the factors that are associated with disease progression. There have been limited previous studies with small population sizes about the systemic progression of GPA localized to the respiratory tract, particularly involving the sinonasal passages [14,16]. Our objectives for this study were to compare disease progression and disease activity in LGPA patients with and without a diagnosis of CRS. Furthermore, we wanted to determine whether patients that undergo functional endoscopic sinus surgery (FESS) who are postulated to have more severe disease are associated with higher risk of disease progression.

## 2. Materials and Methods

We used the US Collaborative Network of the TriNetX platform (Cambridge, MA, USA) to conduct this retrospective study. This platform collects deidentified data from the electronic health record of nearly 100,000,000 patients from over 50 healthcare organizations. This retrospective study is exempt from informed consent. The data reviewed are a secondary analysis of existing data, do not involve intervention or interaction with human subjects, and are deidentified as per the deidentification standard defined in Section §164.514(a) (https://www.ecfr.gov/current/title-45/subtitle-A/subchapter-C/part-164/subpart-E/section-164.514 (accessed on 21 April 2025)). of the HIPAA Privacy Rule. The process by which the data are deidentified is attested to through a formal determination by a qualified expert as defined in Section §164.514(b)(1) of the HIPAA Privacy Rule. This formal determination by a qualified expert refreshed on December 2020. In addition, our study protocol using the TriNetX platform was also approved as exempt human subject research by the Cleveland Clinic Institutional Review Board.

**Figure 1:**
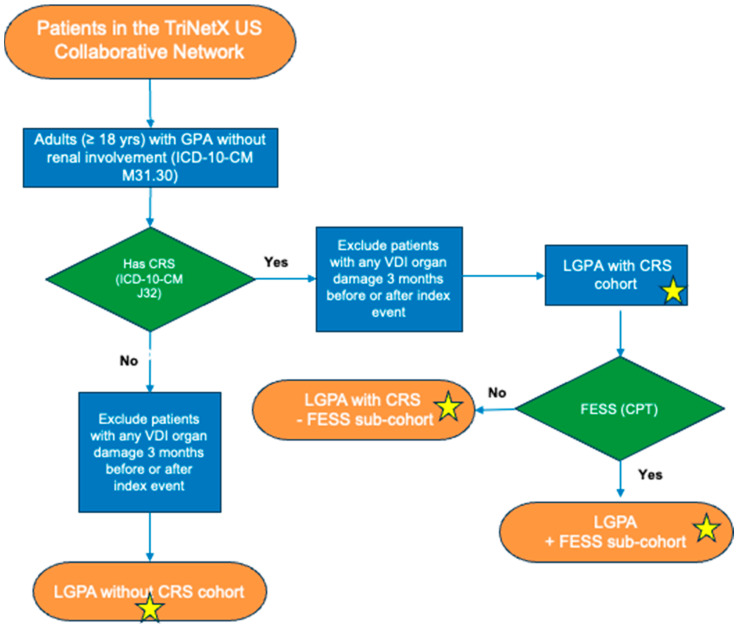
**Cohort and Subcohort Flowchart.** Starred are the cohorts and subcohorts used for analysis.

For our cohort inclusion criteria, we selected adult patients with encounter diagnosis of GPA without renal involvement (that we defined as having LGPA) that also had or did not have a CRS International Classification of Diseases, Tenth Revision, Clinical Modification (ICD-10-CM) encounter diagnosis. Data were obtained as of 15 October 2024. We excluded any patients who had any VDI organ damage 3 months before or after meeting all inclusion criteria. We also defined subcohorts from within the LGPA patients with CRS group of patients who underwent FESS or did not undergo FESS. Figure 1 depicts a flowchart of these cohort and subcohort definitions. Encounter diagnoses were based on ICD-10-CM codes, procedures utilized Current Procedural Terminology (CPT) and/or Systematized Nomenclature of Medicine Clinical Terms (SNOMED) codes, and lab values utilized Logical Observation Identifiers Names and Codes (LOINC) codes. All codes used for each cohort and subcohort are provided in Supplemental Material S1.

We performed a 1:1 propensity match on age, gender, ethnicity, and race. The outcomes evaluated occurred from one month to up to five years after patients met all inclusion criteria (the index event), and patients who had the outcome occur prior to this time window were excluded from analysis. The primary outcomes we evaluated were (1) major disease activity, which was defined by having any “major” organ system disease activity based on the BVAS/WG criteria (not including any non-major organ system disease activity), (2) organ damage, defined by having any end-organ damage based on the VDI criteria, and (3) local sinonasal disease, as indicated by CRS acute exacerbation (acute sinusitis ICD-10-CM code).

The secondary outcomes included organ system breakdown of the end-organ damage and major disease activity primary outcomes, as well as mortality analysis. We evaluated end-organ damage for the following VDI-defined organ systems: musculoskeletal, skin/mucous membranes, ocular, ear/nose/throat (ENT), pulmonary, cardiovascular, peripheral vascular disease, gastrointestinal, renal, neuropsychiatric, and other. We also evaluated BVAS/WG-defined major disease activity for each organ system: renal, cutaneous, eye, ear, GI, respiratory, and brain/spinal cord. Mortality was evaluated by the demographic measure for “deceased”. All codes used for each primary and secondary outcome are also found in Supplemental Material S1.

The statistical analysis was conducted using the built-in TriNetX analytical tools. We compared relative risk between the experimental and control cohorts and subcohorts, using chi-squared tests for categorical variables and independent sample *t*-tests for continuous variables. Furthermore, the TriNetX platform had a built-in logistic regression tool for 1:1 propensity matching.

## 3. Results

Prior to a 1:1 propensity match, there were a total of 1254 patients in the LGPA with CRS cohort and 3534 patients in the LGPA without CRS cohort. After a 1:1 propensity match, there were 1023 patients in each of the cohorts (LGPA with CRS and LGPA without CRS). For the subcohorts of LGPA with CRS who underwent FESS and did not undergo FESS, there were 427 patients in each group after propensity matching. Table 1 shows the total patient count before and after propensity matching for cohorts and subcohorts. The mean age for LGPA patients with CRS was 51.8 ± 16.6, and the mean age for LGPA patients without CRS was 51.9 ± 16.9 after propensity matching, with no significant difference. There were 59.9% females and 38.2% males in the cohort of LGPA with CRS patients and there were 59.6% females and 38.4% males in the cohort of LGPA without CRS patients, with no significant difference of gender between these two cohorts. Demographic characteristics before and after propensity matching for the main cohorts are found in Table 2.

Patients with LGPA and CRS were more likely to have acute sinusitis, end-organ damage, and major disease activity than those without CRS. Table 3 outlines these primary outcomes. For secondary outcomes, we determined the breakdown of organ system involvement in VDI-defined end-organ damage (Table 4) and BVAS/WG-defined major organ system activity (Table 5). We found that all organ systems showed greater end-organ damage in CRS patients than non-CRS patients, except for the gastrointestinal system, which had too few patients for analysis. Importantly, the organs with the highest risk of being affected in CRS patients were cardiovascular and ENT end-organ damage. For major disease activity, we found that ear and pulmonary systems had more disease activity in patients with CRS than those without CRS.

Despite experiencing more progression to organ damage and major disease activity, there was no difference in death in within the follow-up period in LGPA patients with CRS compared to LGPA patients without CRS (risk ratio: 0.94, 95% CI [0.64,1.38]).

We performed subgroup analysis on LGPA patients with CRS and compared those who underwent FESS vs. those who did not. There was no significant difference in local sinonasal disease exacerbations (as indicated by acute sinusitis), end-organ damage, or major disease activity. Results are shown in Table 6.

The breakdown of organ system end-organ damage for this subgroup analysis of patients with LGPA and CRS with or without FESS (Table 7) showed that there was significantly more ENT end-organ damage (which includes ENT diseases other than CRS) and less renal end-organ damage in patients who underwent FESS. While not statistically significant, risk of end-organ damage was lower in all other organ systems, except for ENT, pulmonary, and neuropsychiatric systems, in those who underwent FESS compared to those who did not. There was greater risk of ear major disease activity but less risk of pulmonary major disease activity in patients who underwent FESS compared to those who did not undergo FESS, as shown in Table 8. Finally, there was no significant difference in mortality for patients who underwent FESS compared to those who did not (risk ratio: 1.16, 95% CI [0.62,2.16], *p* = 0.629).

## 4. Discussion

Our study is the largest to date looking at the relationship between sinonasal and systemic exacerbations of patients with LGPA and CRS. A few previous articles have studied the systemic progression of patients with LGPA and head and neck or airway manifestations [14,16,17], but none, to our knowledge, have compared those with CRS to those without CRS.

Those previous studies have suggested systemic disease progression to be possible in patients with LGPA and head and neck or airway manifestations, although rare compared to pulmonary and ENT disease manifestations [14,16,17]. For example, Holle et al. found that only 10% of patients progressed to systemic disease in their 50 patients with LGPA. They found that over 95% of their patients presented with CRS at disease onset and that 66% of their patients had end-organ damage (VDI score > 0) at the end of the follow-up period (median follow-up 48 months) and were mostly ENT manifestations [14]. Taylor et al. conducted a retrospective study of 24 GPA patients and found that two out of 13 patients (15.38%) who exhibited only head and neck manifestations of GPA progressed to other organ disease, specifically pulmonary manifestations but no renal disease [16]. These studies align with our results, as we found that patients with LGPA with CRS experienced more sinonasal exacerbations and a higher progression to end-organ damage and major disease activity compared to those without CRS. This relationship was consistent in our analysis of secondary outcomes related to end-organ damage and major disease activity across all organ systems with a sufficiently large sample size. Notably, we observed the highest percentage of end-organ damage in the ENT (15.30% of CRS patients) and pulmonary (14.57% of CRS patients) systems, the highest risk of damage in the ENT and cardiovascular (in which we have included the ICD code for “Other specified symptoms and signs involving the circulatory and respiratory systems”, which therefore may include pulmonary manifestations) systems, and the highest risk of major disease activity in ear and pulmonary systems in patients with LGPA with CRS compared to without CRS.

Interestingly, two previous studies conducted by the same study group have described the absence of ENT manifestations to be associated with greater mortality risk [18,19]. Meanwhile, we found no difference in mortality risk in LGPA patients without CRS compared to those with CRS. However, our study differs from previous studies in that we only studied the absence of CRS rather than the absence of all ENT manifestations. The previous studies have posited that ENT manifestations may be indicative of a more localized benign granulomatous [18,19].

In our study, we found that LGPA patients with CRS who underwent FESS had more ENT-related organ damage beyond just CRS, such as hearing loss, subglottic stenosis, and/or septal perforation. A longitudinal observational study by Holme et al. looked at 127 patients with GPA, comparing those who had FESS with those who did not [20]. Although Holme et al. studied all GPA patients, not just those with LGPA, they found that patients who underwent FESS had a higher prevalence, severity, and progression rate of sinus osteitis, as seen on CT scans, compared to those who did not have sinus surgery. They suggested that GPA patients referred for FESS might have more severe sinonasal disease [20]. While we did not specifically look at sinus osteitis, our findings suggest that LGPA patients referred for FESS may also have more severe ENT issues beyond just sinonasal disease.

In clinical practice, physicians may worry that FESS signals the progression of a patient’s GPA. However, our results suggest that FESS does not necessarily indicate systemic progression of the disease as there was lower risk of renal end-organ damage and pulmonary major disease activity compared to those who did not undergo FESS. Meanwhile, FESS may reflect more severe local ENT involvement. Additionally, our study emphasizes the importance of close collaboration between otolaryngology and rheumatology in managing patients with CRS and GPA. A multidisciplinary approach to care could improve disease management, addressing the multisystem effects of GPA. More research is needed to better understand the underlying pathophysiology and why CRS may serve as an indicator of more systemic progression.

The strengths of our study include its very large sample size. However, there are several limitations. First, the retrospective nature of the study is a limitation. We were also unable to determine the severity of CRS, as our data did not allow for a precise assessment of this. Furthermore, there were no subjective measures of sinonasal outcomes (e.g., Sino-nasal Outcome Test-22) available on the TriNetX platform. Additionally, we relied on ICD codes entered as encounter diagnoses, so we cannot be completely certain of the accuracy of the diagnoses. While FESS was used as a proxy for CRS severity, our dataset did not provide detailed information on CRS severity. We also could not determine individual patient VDI and BVAS/WG scores, which would have been helpful for quantifying systemic disease severity and activity. Some criteria for major disease in BVAS/WG or VDI-defined end-organ damage could not be adequately captured through the available codes in the TriNetX platform. For example, the VDI includes factors like estimated or measured GFR ≤ 50%, but when there was no specific code for this, we had to use codes that most closely represented the factor. Additionally, we could not obtain information about the indications for FESS, nor could we determine the immunosuppressive regimen that patients were receiving for their GPA, due to limitations of the TriNetX platform. Lastly, the mortality data from the TriNetX aggregated electronic health records may not be fully accurate, as many deaths occur outside healthcare organizations and are not always documented in electronic health records.

## 5. Conclusions

In patients with LGPA and CRS, there is a higher risk of disease progression and increased activity across organ systems compared to those with LGPA alone. The most significant progression occurs in ENT damage. Patients who underwent FESS exhibited the highest major disease activity in the ear and ENT organ damage but lower risk of renal damage and pulmonary disease activity compared to those who never had FESS.

## Figures and Tables

**Table 1:** Cohort population sizes before and after 1:1 propensity matching.

Cohort	Before Matching	After Matching
CRS		
LGPA + CRS	1254	1162
LGPA − CRS	3534	1162
FESS		
LGPA + CRS + FESS	568	534
LGPA + CRS − FESS	815	534

CRS: chronic rhinosinusitis, FESS: functional endoscopic sinus surgery, LGPA: limited granulomatosis with polyangiitis.

**Table 2:** Demographics of LGPA with and without CRS before and after propensity matching.

Characteristic	Before Matching				After Matching			
	LGPA + CRS (N = 1254)	LGPA − CRS (N = 3534)	*p*-Value	SMD	LGPA + CRS (N = 1162)	LGPA − CRS (N = 1162)	*p*-Value	SMD
Age at Index (Mean ± SD)	51.8 ± 16.6	52.6 ± 18.0	0.151	0.050	51.8 ± 16.6	51.9 ± 16.9	0.821	0.009
Gender								
Male	38.2%	40.3%	0.202	0.044	38.2%	38.4%	0.932	0.004
Female	59.9%	57.1%	0.101	0.056	59.9%	59.6%	0.899	0.005
Unknown Gender	1.9%	2.5%	0.223	0.043	1.9%	2.0%	0.880	0.006
Race								
White	77.7%	71.1%	<0.001	0.152	77.7%	79.9%	0.187	0.055
Black or African American	5.3%	4.2%	0.105	0.054	5.3%	4.3%	0.245	0.048
Asian	1.8%	2.3%	0.314	0.035	1.8%	1.1%	0.167	0.057
American Indian or Alaska Native	0.9%	0.5%	0.160	0.045	0.9%	0.9%	1	<0.001
Native Hawaiian or Pacific Islander	0.9%	0.3%	0.016	0.073	0.9%	0.9%	1	<0.001
Other Race	3.7%	5.0%	0.073	0.063	3.7%	2.9%	0.297	0.043
Ethnicity								
Hispanic or Latino	7.3%	6.2%	0.182	0.045	7.3%	6.2%	0.283	0.045
Not Hispanic or Latino	70.8%	65.4%	0.001	0.116	70.8%	71.8%	0.614	0.021

CRS: chronic rhinosinusitis, LGPA: limited granulomatosis with polyangiitis, SMD: standardized mean difference.

**Table 3:** Primary outcomes of patients with LGPA ± CRS.

Cohort	CRS	No CRS	Risk Ratio [95% CI]	*p*-Value
Acute Sinusitis	72/946 (7.61)	18/1136 (1.58)	4.80 [2.89, 7.99]	**<0.001**
End-Organ Damage	140/335 (41.8)	133/874 (15.2)	2.75 [2.25, 3.36]	**<0.001**
Major Disease Activity	102/899 (11.35)	49/1040 (4.71)	2.41 [1.73, 3.35]	**<0.001**

CI: confidence interval, CRS: chronic rhinosinusitis, LGPA: limited granulomatosis with polyangiitis. Data are presented as n/ Cohort N included in results (%). Patients were excluded from the total Cohort N if they had the outcome outside of the 1-month-5-year time window.

**Table 4:** Secondary outcomes of end-organ damage (VDI) patients with LGPA ± CRS.

End-Organ Damage (VDI)	CRS	No CRS	Risk Ratio [95% CI]	*p*-Value
Musculoskeletal	69/1055 (6.54)	31/1126 (2.75)	2.38 [1.57, 3.60]	**<0.001**
Skin/mucous membranes	28/1115 (2.51)	* n/a /1149	** 2.89 [1.41, 5.91]	** 0.002
Ocular	44/1090 (4.04)	20/1136 (1.76)	2.29 [1.36, 3.86]	**0.001**
ENT	103/673 (15.30)	34/1067 (3.19)	4.80 [3.30, 6.99]	**<0.001**
Pulmonary	118/810 (14.57)	51/1045 (4.88)	2.99 [2.18, 4.09]	**<0.001**
Cardiovascular	100/978 (10.22)	32/1122 (2.85)	3.59 [2.43, 5.29]	**<0.001**
Peripheral vascular disease	49/1086 (4.51)	26/1127 (2.31)	1.96 [1.26, 3.12]	**0.004**
Gastrointestinal	* n/a /1147	* n/a /1155	* n/a	* n/a
Renal	51/948 (5.38)	19/1010 (1.88)	2.86 [1.70, 4.81]	**<0.001**
Neuropsychiatric	41/926 (4.43)	16/990 (1.62)	2.74 [1.55, 4.85]	**<0.001**
Other	89/828 (10.75)	62/960 (6.46)	1.66 [1.22, 2.27]	**0.001**

CI: confidence interval, CRS: chronic rhinosinusitis, ENT: ear, nose, and throat, LGPA: limited granulomatosis with polyangiitis, VDI: Vasculitis Disease Index. Data are presented as n/ Cohort N included in results (%). * n/a means sample was too small to analyze (between 1 and 10). ** Risk ratio and *p*-value based on using value of 10 for * n/a. Patients were excluded from the total Cohort N if they had the outcome outside of the 1-month-5-year time window.

**Table 5:** Secondary outcomes of major disease activity by organ system (BVAS/WG) patients with LGPA ± CRS.

Major Disease Activity by Organ System (BVAS/WG)	FESS	No FESS	Risk Ratio [95% CI]	*p*-Value
Cutaneous	* n/a /1026	0	* n/a	* n/a
Eye	17/1128 (1.51)	* n/a /1133	** 1.71 [0.79, 3.71]	** 0.172
Ear	33/1114 (2.96)	* n/a /1148	** 3.40 [1.68, 6.87]	** <0.001
Gastrointestinal	* n/a /1158	* n/a /1160	* n/a	* n/a
Pulmonary	49/1049 (4.67)	19/1110 (1.71)	2.73 [1.62, 4.60]	**<0.001**
Renal	* n/a /1154	* n/a /1160	* n/a	* n/a
Nervous	51/1027 (4.97)	37/1105 (3.35)	1.48 [0.98, 2.25]	0.061

BVAS/WG: Birmingham Vasculitis Activity Score for Wegener’s Granulomatosis, CI: confidence interval, CRS: chronic rhinosinusitis, LGPA: limited granulomatosis with polyangiitis. Data are presented as n/ Cohort N included in results (%). * n/a means sample was too small to analyze (between 1 and 10). ** Risk ratio and *p*-value based on using value of 10 for * n/a. Patients were excluded from the total Cohort N if they had the outcome outside of the 1-month-5-year time window.

**Table 6:** Primary outcomes of patients with LGPA + CRS ± FESS.

Cohort	FESS	No FESS	Risk Ratio [95% CI]	*p*-Value
Acute sinusitis	37/408 (9.07)	30/429 (6.99)	1.30 [0.82, 2.06]	0.269
End-organ Damage	43/98 (43.88)	62/162 (38.27)	1.15 [0.85, 1.54]	0.372
Major disease activity	55/429 (12.82)	46/389 (11.83)	1.08 [0.75, 1.57]	0.666

CI: confidence interval, CRS: chronic rhinosinusitis, FESS: functional endoscopic sinus surgery, LGPA: limited granulomatosis with polyangiitis. Data are presented as n/Cohort N included in results (%). Patients were excluded from the total Cohort N if they had the outcome outside of the 1-month-5-year time window.

**Table 7:** Secondary outcomes of end-organ damage (VDI) patients with LGPA ± CRS ± FESS.

End-Organ Damage (VDI)	FESS	No FESS	Risk Ratio [95% CI]	*p*-Value
Musculoskeletal	33/478 (6.90)	35/485 (7.22)	0.96 [0.61, 1.51]	0.850
Skin/mucous membranes	11/505 (2.18)	16/509 (3.14)	0.69 [0.33, 1.48]	0.340
Ocular	15/491 (3.05)	21/504 (4.17)	0.73 [0.38, 1.41]	0.348
ENT	38/176 (21.59)	40/366 (10.93)	1.98 [1.32, 2.97]	**0.001**
Pulmonary	62/370 (16.76)	51/356 (14.33)	1.17 [0.83, 1.65]	0.366
Cardiovascular	40/431 (9.28)	51/451 (11.31)	0.82 [0.55, 1.22]	0.322
Peripheral vascular disease	15/500 (3.00)	27/496 (5.44)	0.55 [0.30, 1.02]	0.055
Gastrointestinal	* n/a /528	* n/a /527	* n/a	* n/a
Renal	21/491 (4.28)	36/489 (7.36)	0.58 [0.34, 0.98]	**0.039**
Neuropsychiatric	34/490 (6.94)	21/474 (4.43)	1.57 [0.92, 2.66]	0.093
Other	43/435 (9.89)	44/406 (10.84)	0.91 [0.61, 1.36]	0.650

CI: confidence interval, CRS: chronic rhinosinusitis, ENT: ear, nose, and throat, FESS: functional endoscopic sinus surgery, VDI: vasculitis disease index. Data are presented as n/ Cohort N included in results (%). * n/a means sample was too small to analyze (between 1 and 10). Patients were excluded from the total Cohort N if they had the outcome outside of the 1-month-5-year time window.

**Table 8:** Secondary outcomes of major disease activity by organ system (BVAS/WG) patients with LGPA ± CRS ± FESS.

Major Disease Activity by Organ System (BVAS/WG)	FESS	No FESS	Risk Ratio [95% CI]	*p*-Value
Cutaneous	* n/a /534	* n/a /532	* n/a	* n/a
Eye	* n/a /523	* n/a /514	* n/a	* n/a
Ear	29/503 (5.77)	11/514 (2.14)	2.69 [1.36, 5.33]	**0.003**
Gastrointestinal	* n/a /532	* n/a /533	* n/a	* n/a
Pulmonary	13/490 (2.65)	26/464 (5.60)	0.47 [0.25, 0.91]	**0.021**
Renal	* n/a /529	0	* n/a	* n/a
Nervous	34/469 (7.25)	28/469 (5.97)	1.21 [0.75, 1.97]	0.430

BVAS/WG: Birmingham Vasculitis Activity Score for Wegener’s Granulomatosis, CI: confidence interval, CRS: chronic rhinosinusitis, FESS: functional endoscopic sinus surgery, VDI: vasculitis disease index. Data are presented as n/ Cohort N included in results (%). * n/a means sample was too small to analyze (between 1 and 10). Patients were excluded from the total Cohort N if they had the outcome outside of the 1-month-5-year time window.

## Data Availability

Data are propriety to the TriNetX platform.

## Supplementary Materials

The following supporting information can be downloaded at: https://www.mdpi.com/article/10.3390/jcm14092962/s1, Supplemental Material S1: Codes used for cohorts, subcohorts, and outcomes.

## Abbreviations

The following abbreviations are used in this manuscript:

BVAS/WG	Birmingham Vasculitis Activity Score for Wegener’s Granulomatosis
CPT	Current Procedural Terminology
CRS	Chronic rhinosinusitis
ENT	Ear/nose/throat
FESS	Functional endoscopic sinus surgery
GPA	Granulomatosis with polyangiitis
ICD-10-CM	International Classification of Diseases, Tenth Revision, Clinical Modification
LGPA	Limited granulomatosis with polyangiitis
LOINC	Logical Observation Identifiers Names and Codes
SNOMED	Systematized Nomenclature of Medicine Clinical Terms
VDI	Vasculitis Disease Index
