# Supplementary material for: Impact of Chronic Rhinosinusitis on Granulomatosis with Polyangiitis Exacerbations
Source: J Clin Med. 2025 Apr 25;14(9):2962. doi: 10.3390/jcm14092962 (PMC12073045; doi:10.3390/jcm14092962)
Supplement: Supplementary file 1 [file jcm-14-02962-s001.zip › jcm-3553324-supplementary.pdf]

Supplemental Material S1. Codes used for cohorts, sub-cohorts, and outcomes.

Cohorts and Subcohorts

1. Limited granulomatosis with polyangiitis and chronic rhinosinusitis cohort

Patients were defined as having limited granulomatosis with polyangiitis and chronic rhinosinusitis if they met the following groups of conditions:

a. Adults (≥ 18 years old)
b. Group A- Must have:
    i. ICD10CM:J32 “Chronic Sinusitis”
    ii. ICD10CM:M31.30 “Wegener's granulomatosis without renal involvement”
c. Group B- Any instance of the following diagnoses cannot have occurred within 3 months before or up to 3 months after any instance of Group A (based on the Vasculitis Damage Index).
    i. Musculoskeletal
        1. ICD10CM:M62.5 “Muscle wasting and atrophy, not elsewhere classified”
        2. ICD10CM:M15.4 “Erosive (osteo)arthritis”
        3. ICD10CM:M80 “Osteoporosis with current pathological fracture”
        4. ICD10CM:M81 “Osteoporosis without current pathological fracture”
        5. ICD10CM:M87 “Osteonecrosis”
        6. ICD10CM:M86 “Osteomyelitis”
        7. ICD10CM:M95 “Other acquired deformities of musculoskeletal system and connective tissue”
    ii. Skin/ Mucous membranes
        1. ICD10CM:K13.7 “Other and unspecified lesions of oral mucosa”
        2. ICD10CM:L65 “Other nonscarring hair loss”
        3. ICD10CM:L98.49 “Non-pressure chronic ulcer of skin of other sites”
    iii. Ocular
        1. ICD10CM:H54 “Blindness and low vision”
        2. ICD10CM:H53.2 “Diplopia”
        3. ICD10CM:H47.2 “Optic atrophy”
        4. ICD10CM:H35 “Other retinal disorders”
        5. ICD10CM:H26 “Other cataract”
        6. ICD10CM:S02.83 “Fracture of medial orbital wall”
        7. ICD10CM:S02.84 “Fracture of lateral orbital wall”
        8. ICD10CM:S02.85 “Fracture of orbit, unspecified”
    iv. Ear, Nose, Throat
        1. ICD10CM:H90.3 “Sensorineural hearing loss, bilateral”
        2. ICD10CM:H90.4 “Sensorineural hearing loss, unilateral with unrestricted hearing on the contralateral side”
        3. ICD10CM:H91 “Other and unspecified hearing loss”
        4. ICD10CM:H90.5 “Unspecified sensorineural hearing loss”

    5. ICD10CM:M95.0 “Acquired deformity of nose”
    6. CPT:30630 “Repair nasal septal perforations”
    7. SNOMED:63145008 “Repair of nasal septal perforation”
    8. ICD10PCS:09QM3ZZ “Repair Nasal Septum, Percutaneous Approach”
    9. ICD10PCS:09QM4ZZ “Repair Nasal Septum, Percutaneous Endoscopic Approach”
    10. ICD10PCS:09QM0ZZ “Repair Nasal Septum, Open Approach”
    11. ICD10CM:J38.6 “Stenosis of larynx”

v. Pulmonary
    1. ICD10CM:I27.2 “Other secondary pulmonary hypertension”
    2. ICD10CM:I27.0 “Primary pulmonary hypertension”
    3. ICD10CM:J84.10 “Pulmonary fibrosis, unspecified”
    4. ICD10CM:I26 “Pulmonary embolism”
    5. ICD10CM:J45 “Asthma”
    6. ICD10CM:R06.0 “Dyspnea”

vi. Cardiovascular
    1. ICD10CM:Z95.5 “Presence of coronary angioplasty implant and graft”
    2. ICD10CM:I21 “Acute myocardial infarction”
    3. ICD10CM:I42 “Cardiomyopathy”
    4. ICD10CM:I50 “Heart Failure”
    5. ICD10CM:I31 “Other diseases of pericardium”
    6. ICD10CM:I32 “Pericarditis in diseases classified elsewhere”
    7. LNC:96609-3 “Diastolic blood pressure mean (at least 95.00 mm[Hg])”
    8. ICD10CM:R09.8 “Other specified symptoms and signs involving the circulatory and respiratory systems”

vii. Peripheral Vascular Disease
    1. ICD10CM:I73 “Other peripheral vascular diseases”
    2. ICD10CM:M79.9 “Soft tissue disorder, unspecified”
    3. ICD10CM:I82 “Other venous embolism and thrombosis”
    4. ICD10CM:I65 “Occlusion and stenosis of precerebral arteries, not resulting in cerebral infarction”
    5. ICD10CM:M62.2 “Nontraumatic ischemic infarction of muscle”

viii. Gastrointestinal
    1. ICD10CM:K55.0 “Acute vascular disorders of intestine”
    2. ICD10CM:K55.1 “Chronic vascular disorders of intestine”
    3. ICD10CM:K86.1 “Other chronic pancreatitis”
    4. ICD10CM:K65.8 “Other peritonitis”
    5. ICD10CM:K22.2 “Esophageal obstruction”

ix. Renal
    1. ICD10CM:N05 “Unspecified nephritic syndrome”

2. ICD10CM:M31.31 “Wegener's granulomatosis with renal involvement”
3. LNC:2889-4 “Protein [Mass/time] in 24 hour Urine (at least 0.50 g/(24.h))”
4. ICD10CM:N18.6 “End stage renal disease”

x. Neuropsychiatric
1. ICD10CM:G31.84 “Mild cognitive impairment of uncertain or unknown etiology”
2. ICD10CM:F29 “Unspecified psychosis not due to a substance or known physiological condition”
3. ICD10CM:G40 “Epilepsy and recurrent seizures”
4. ICD10CM:R56 “Convulsions, not elsewhere classified”
5. ICD10CM:I63 “Cerebral infarction”
6. ICD10CM:G52 “Disorders of other cranial nerves”
7. ICD10CM:S04 “Injury of cranial nerve”
8. ICD10CM:G56 “Mononeuropathies of upper limb”
9. ICD10CM:G60.3 “Idiopathic progressive neuropathy”
10. ICD10CM:G58 “Other mononeuropathies”
11. ICD10CM:G59 “Mononeuropathy in diseases classified elsewhere”
12. ICD10CM:G57 “Mononeuropathies of lower limb”

xi. Other
1. ICD10CM:E28.8 “Other ovarian dysfunction”
2. ICD10CM:E28.39 “Other primary ovarian failure”
3. ICD10CM:E29.1 “Testicular hypofunction”
4. ICD10CM:D60-D64 “Aplastic and other anemias and other bone marrow failure syndromes”
5. ICD10CM:E08-E13 “Diabetes Mellitus”
6. ICD10CM:N30 “Cystitis”
7. ICD10CM:C80 “Malignant neoplasm without specification of site”
8. ICD10CM:G04.89 “Other myelitis”

2. Limited granulomatosis with polyangiitis and without chronic rhinosinusitis cohort

Patients were defined as having limited granulomatosis with polyangiitis and without chronic rhinosinusitis if they met the following groups of conditions:

a. Adults (≥ 18 years old)

b. Group A

i. Must have:
1. ICD10CM:M31.30 “Wegener's granulomatosis without renal involvement”

ii. Cannot have:
1. ICD10CM:J32 “Chronic Sinusitis”

c. Group B- Same as Group B in the “Limited granulomatosis with polyangiitis and chronic rhinosinusitis cohort” listed above

3. Limited granulomatosis with polyangiitis with chronic rhinosinusitis patients who underwent functional endoscopic sinus surgery subcohort

Patients in this subcohort were those that met the same criteria as in 1. “Limited granulomatosis with polyangiitis with chronic rhinosinusitis patients” cohort, and also underwent functional endoscopic sinus surgery. They had to have had at least one of the following codes:

   a. CPT:31231 “Nasal endoscopy, diagnostic, unilateral or bilateral (separate procedure)”
   b. CPT:31233 “Nasal/sinus endoscopy, diagnostic; with maxillary sinusoscopy (via inferior meatus or canine fossa puncture)”
   c. CPT:31235 “Nasal/sinus endoscopy, diagnostic; with sphenoid sinusoscopy (via puncture of sphenoidal face or cannulation of ostium)”
   d. CPT:31237 “Nasal/sinus endoscopy, surgical; with biopsy, polypectomy or debridement (separate procedure)”
   e. CPT:31238 “Nasal/sinus endoscopy, surgical; with control of nasal hemorrhage”
   f. CPT:31295 “Nasal/sinus endoscopy, surgical, with dilation (eg, balloon dilation); maxillary sinus ostium, transnasal or via canine fossa”
   g. CPT:31296 “Nasal/sinus endoscopy, surgical, with dilation (eg, balloon dilation); frontal sinus ostium”
   h. CPT:31297 “Nasal/sinus endoscopy, surgical, with dilation (eg, balloon dilation); sphenoid sinus ostium“
   i. CPT:31254 “Nasal/sinus endoscopy, surgical with ethmoidectomy; partial (anterior)”
   j. CPT:31255 “Nasal/sinus endoscopy, surgical with ethmoidectomy; total (anterior and posterior)”
   k. CPT:31276 “Nasal/sinus endoscopy, surgical, with frontal sinus exploration, including removal of tissue from frontal sinus, when performed”
   l. CPT:31256 “Nasal/sinus endoscopy, surgical, with maxillary antrostomy”
   m. CPT:31267 “Nasal/sinus endoscopy, surgical, with maxillary antrostomy; with removal of tissue from maxillary sinus”
   n. CPT:31287 “Nasal/sinus endoscopy, surgical, with sphenoidotomy”
   o. CPT:31288 “Nasal/sinus endoscopy, surgical, with sphenoidotomy; with removal of tissue from the sphenoid sinus”

4. Limited granulomatosis with polyangiitis with chronic rhinosinusitis patients who did not undergo functional endoscopic sinus surgery subcohort

Patients in this subcohort were those that met the same criteria as in 1. “Limited granulomatosis with polyangiitis with chronic rhinosinusitis patients” cohort, and also did not ever undergo functional endoscopic sinus surgery. They could not have had any of the codes that were listed in 3a-o.

Outcomes

Patients had the following outcomes within 1 month to 5 years after meeting all inclusion criteria.

1. ICD10CM:J01 “Acute Sinusitis”
2. Organ Damage (based on VDI)
   a. Musculoskeletal

    i. ICD10CM:M62.5 “Muscle wasting and atrophy, not elsewhere classified”; or
    ii. ICD10CM:M15.4 “Erosive (osteo)arthritis”; or
    iii. ICD10CM:M80 “Osteoporosis with current pathological fracture”; or
    iv. ICD10CM:M81 “Osteoporosis without current pathological fracture”; or
    v. ICD10CM:M87 “Osteonecrosis”; or
    vi. ICD10CM:M86 “Osteomyelitis”; or
    vii. ICD10CM:M95 “Other acquired deformities of musculoskeletal system and connective tissue”
b. Skin/ Mucous membranes
    i. ICD10CM:K13.7 “Other and unspecified lesions of oral mucosa”; or
    ii. ICD10CM:L65 “Other nonscarring hair loss”; or
    iii. ICD10CM:L98.49 “Non-pressure chronic ulcer of skin of other sites”
c. Ocular
    i. ICD10CM:H54 “Blindness and low vision”; or
    ii. ICD10CM:H53.2 “Diplopia”; or
    iii. ICD10CM:H47.2 “Optic atrophy”; or
    iv. ICD10CM:H35 “Other retinal disorders”; or
    v. ICD10CM:H26 “Other cataract”; or
    vi. ICD10CM:S02.8 “Fracture of medial orbital wall”; or
    vii. ICD10CM:S02.84 “Fracture of lateral orbital wall”; or
    viii. ICD10CM:S02.85 “Fracture of orbit, unspecified”
d. Ear, Nose, Throat
    i. ICD10CM:H90.3 “Sensorineural hearing loss, bilateral”; or
    ii. ICD10CM:H90.4 “Sensorineural hearing loss, unilateral with unrestricted hearing on the contralateral side”; or
    iii. ICD10CM:H91 “Other and unspecified hearing loss”; or
    iv. ICD10CM:H90.5 “Unspecified sensorineural hearing loss”; or
    v. CPT:30630 “Repair nasal septal perforations”; or
    vi. SNOMED:63145008 “Repair of nasal septal perforation”; or
    vii. ICD10PCS:09QM0ZZ “Repair Nasal Septum, Open Approach”; or
    viii. ICD10PCS:09QM3ZZ “Repair Nasal Septum, Percutaneous Approach”; or
    ix. ICD10PCS:09QM4ZZ “Repair Nasal Septum, Percutaneous Endoscopic Approach”; or
    x. ICD10CM:J34 “Other and unspecified disorders of nose and nasal sinuses”; or
    xi. ICD10CM:J38.6 “Stenosis of larynx”
e. Pulmonary
    i. ICD10CM:I27.2 “Other secondary pulmonary hypertension”; or
    ii. ICD10CM:I27.0 “Primary pulmonary hypertension”; or
    iii. ICD10CM:J84.10 “Pulmonary fibrosis, unspecified”; or
    iv. ICD10CM:I26 “Pulmonary embolism”; or
    v. ICD10CM:J45 “Asthma”; or

    vi. ICD10CM:R06.0 “Dyspnea”; or

f. Cardiovascular
    i. ICD10CM:Z95.5 “Presence of coronary angioplasty implant and graft”; or
    ii. ICD10CM:I21 “Acute myocardial infarction”; or
    iii. ICD10CM:I42 “Cardiomyopathy”; or
    iv. ICD10CM:I50 “Heart failure”; or
    v. ICD10CM:I31 “Other diseases of pericardium”; or
    vi. ICD10CM:I32 “Pericarditis in diseases classified elsewhere”; or
    vii. LNC:96609-3 “Diastolic blood pressure mean (at least 95.00 mm[Hg] (most recent occurrence))”; or
    viii. ICD10CM:R09.8 “Other specified symptoms and signs involving the circulatory and respiratory systems”; or

g. Peripheral Vascular Disease
    i. ICD10CM:I73 “Other peripheral vascular diseases”; or
    ii. ICD10CM:M79.9 “Soft tissue disorder, unspecified”; or
    iii. ICD10CM:I82 “Other venous embolism and thrombosis”; or
    iv. ICD10CM:I65 “Occlusion and stenosis of precerebral arteries, not resulting in cerebral infarction”; or
    v. ICD10CM:M62.2 “Nontraumatic ischemic infarction of muscle”; or

h. Gastrointestinal
    i. ICD10CM:K55.0 “Acute vascular disorders of intestine”; or
    ii. ICD10CM:K55.1 “Chronic vascular disorders of intestine”; or
    iii. ICD10CM:K86.1 “Other chronic pancreatitis”; or
    iv. ICD10CM:K22.2 “Esophageal obstruction”; or

i. Renal
    i. ICD10CM:N05 “Unspecified nephritic syndrome”; or
    ii. ICD10CM:M31.31 “Wegener's granulomatosis with renal involvement”; or
    iii. LNC:2889-4 “Protein [Mass/time] in 24 hour Urine (at least 0.50 g/(24.h) (most recent occurrence))”; or
    iv. ICD10CM:N18.6 “End stage renal disease”.

j. Neuropsychiatric
    i. ICD10CM:G31.84 “Mild cognitive impairment of uncertain or unknown etiology”; or
    ii. ICD10CM:F29 “Unspecified psychosis not due to a substance or known physiological condition”; or
    iii. ICD10CM:G40 “Epilepsy and recurrent seizures”; or
    iv. ICD10CM:R56 “Convulsions, not elsewhere classified”; or
    v. ICD10CM:I63 “Cerebral infarction”; or
    vi. ICD10CM:G52 “Disorders of other cranial nerves”; or
    vii. ICD10CM:S04 “Injury of cranial nerve”; or
    viii. ICD10CM:G56 “Mononeuropathies of upper limb”; or
    ix. ICD10CM:G60.3 “Idiopathic progressive neuropathy”; or
    x. ICD10CM:G58 “Other mononeuropathies”; or
    xi. ICD10CM:G59 “Mononeuropathy in diseases classified elsewhere”; or

   xii. ICD10CM:G57 “Mononeuropathies of lower limb”; or
   xiii. ICD10CM:G04.89 “Other myelitis”; or
k. Other
   i. ICD10CM:E28.8 “Other ovarian dysfunction”; or
   ii. ICD10CM:E28.39 “Other primary ovarian failure”; or
   iii. ICD10CM:E29.1 “Testicular hypofunction”; or
   iv. ICD10CM:D60-D64 “Aplastic and other anemias and other bone marrow failure syndromes”; or
   v. ICD10CM:E08-E13 “Diabetes mellitus”; or
   vi. ICD10CM:N30 “Cystitis”; or
   vii. ICD10CM:C80 “Malignant neoplasm without specification of site”

3. Major Disease Activity (based on BVAS/WG)
   a. Cutaneous
      i. ICD10CM:I96 “Gangrene, not elsewhere classified”
   b. Eye
      i. ICD10CM:H15.0 “Scleritis”; or
      ii. ICD10CM:H35.6 “Retinal hemorrhage”; or
      iii. ICD10CM:H35.89 “Other specified retinal disorders”
   c. Ear
      i. ICD10CM:H90.5 “Unspecified sensorineural hearing loss”; or
      ii. ICD10CM:H90.3 “Sensorineural hearing loss, bilateral”; or
      iii. ICD10CM:H90.4 “Sensorineural hearing loss, unilateral with unrestricted hearing on the contralateral side”
   d. Gastrointestinal
      i. ICD10CM:K55.9 “Vascular disorder of intestine, unspecified”; or
      ii. ICD10CM:K55.0 “Acute vascular disorders of intestine; or
      iii. ICD10CM:K55.1 “Chronic vascular disorders of intestine”
   e. Pulmonary
      i. ICD10CM:J96 “Respiratory failure, not elsewhere classified”; or
      ii. ICD10CM:R04.2 “Hemoptysis”; or
      iii. ICD10CM:R04.8 “Hemorrhage from other sites in respiratory passages”
   f. Renal
      i. LNC:33804-6 “RBC casts [Presence] in Urine sediment by Light microscopy”; or
      ii. LNC:53278-8 “RBC casts [Presence] in Urine by Computer assisted method”; or
      iii. ICD10CM:R94.4 “Abnormal results of kidney function studies”.
   g. Nervous System
      i. ICD10CM:G03 “Meningitis due to other and unspecified causes”; or
      ii. ICD10CM:G95 “Other and unspecified diseases of spinal cord”; or
      iii. ICD10CM:S24 “Injury of nerves and spinal cord at thorax level”; or
      iv. ICD10CM:S14 “Injury of nerves and spinal cord at neck level”; or
      v. ICD10CM:S34 “Injury of lumbar and sacral spinal cord and nerves at abdomen, lower back and pelvis level”; or

vi. ICD10CM:I63 “Cerebral infarction”; or
vii. ICD10CM:G52 “Disorders of other cranial nerves”; or
viii. ICD10CM:G51 “Facial nerve disorders”; or
ix. ICD10CM:G56 “Mononeuropathies of upper limb”; or
x. ICD10CM:G57 “Mononeuropathies of lower limb”; or
xi. ICD10CM:G61 “Inflammatory polyneuropathy”; or
xii. ICD10CM:G58.7 “Mononeuritis multiplex”
